# The roles of epigallocatechin gallate in the tumor microenvironment, metabolic reprogramming, and immunotherapy

**DOI:** 10.3389/fimmu.2024.1331641

**Published:** 2024-01-29

**Authors:** Dongming Li, Donghui Cao, Yuanlin Sun, Yingnan Cui, Yangyu Zhang, Jing Jiang, Xueyuan Cao

**Affiliations:** ^1^ Department of Gastric and Colorectal Surgery, General Surgery Center, The First Hospital of Jilin University, Changchun, China; ^2^ Division of Clinical Epidemiology, The First Hospital of Jilin University, Changchun, China

**Keywords:** epigallocatechin gallate, tumor microenvironment, antitumor immunity, metabolic reprogramming, immunotherapy

## Abstract

Cancer, a disease that modern medicine has not fully understood and conquered, with its high incidence and mortality, deprives countless patients of health and even life. According to global cancer statistics, there were an estimated 19.3 million new cancer cases and nearly 10 million cancer deaths in 2020, with the age-standardized incidence and mortality rates of 201.0 and 100.7 per 100,000, respectively. Although remarkable advancements have been made in therapeutic strategies recently, the overall prognosis of cancer patients remains not optimistic. Consequently, there are still many severe challenges to be faced and difficult problems to be solved in cancer therapy today. Epigallocatechin gallate (EGCG), a natural polyphenol extracted from tea leaves, has received much attention for its antitumor effects. Accumulating investigations have confirmed that EGCG can inhibit tumorigenesis and progression by triggering apoptosis, suppressing proliferation, invasion, and migration, altering tumor epigenetic modification, and overcoming chemotherapy resistance. Nevertheless, its regulatory roles and biomolecular mechanisms in the immune microenvironment, metabolic microenvironment, and immunotherapy remain obscure. In this article, we summarized the most recent updates about the effects of EGCG on tumor microenvironment (TME), metabolic reprogramming, and anti-cancer immunotherapy. The results demonstrated EGCG can promote the anti-cancer immune response of cytotoxic lymphocytes and dendritic cells (DCs), attenuate the immunosuppression of myeloid-derived suppressor cells (MDSCs) and regulatory T cells (Tregs), and inhibit the tumor-promoting functions of tumor-associated macrophages (TAMs), tumor-associated neutrophils (TANs), and various stromal cells including cancer-associated fibroblasts (CAFs), endothelial cells (ECs), stellate cells, and mesenchymal stem/stromal cells (MSCs). Additionally, EGCG can suppress multiple metabolic reprogramming pathways, including glucose uptake, aerobic glycolysis, glutamine metabolism, fatty acid anabolism, and nucleotide synthesis. Finally, EGCG, as an immunomodulator and immune checkpoint blockade, can enhance immunotherapeutic efficacy and may be a promising candidate for antitumor immunotherapy. In conclusion, EGCG plays versatile regulatory roles in TME and metabolic reprogramming, which provides novel insights and combined therapeutic strategies for cancer immunotherapy.

## 1 Introduction

Globally, there were approximately 10 million cancer-related deaths and 19.3 million new cases of cancer in 2020, according to cancer statistics (1–3). With cancer incidence gradually increasing yearly, the worldwide cancer burden is estimated to be close to 28.4 million cases in 2040, up 47% compared to 2020, and this rise is likely to further worsen as the risk factors caused by globalization and economic growth, resulting in even greater healthcare and economic burdens (3).

The tumor microenvironment (TME) and metabolic reprogramming, two notorious pathologic alterations, play pivotal roles in carcinogenesis and progression. It’s commonly known that in addition to malignant tumor cells, TME contains various immune cells, such as lymphocytes and monocytes, as well as various stromal cells, such as fibroblasts and endotheliocytes, all of which have their own unique immunological or immunomodulatory capacity to determine whether a tumor will survive and form complex crosstalk with nearby cells (4). As a shelter for tumor survival, the TME continuously interacts with tumor cells to encourage tumor initiation, invasion, intravasation, metastasis, and treatment resistance (5, 6). Additionally, tumor cells have to spontaneously alter their metabolic flux in TME to maintain the bioenergy and biosynthesis required for their uncontrolled proliferation (7). Thus, the tumor cells rewire their metabolic program known as metabolic reprogramming, driven by the carcinogenic alterations in the tumor cells and the various host cytokines acting on tumor cells in TME (8). These metabolic changes in turn reshape the TME to restrict the immune response and create further obstacles to antitumor treatment (9). Consequently, targeting TME and metabolic reprogramming have been gradually recognized as crucial strategies to remove obstruction to antitumor therapy and immune response in recent years (5, 9, 10).

In fact, novel immunotherapeutic strategies targeting TME, including immune checkpoint blockade have been clinically applied in antitumor therapy (11). Although the rapid rise of immunotherapy has revolutionized the anti-cancer therapeutic landscape in the last decade, clinical outcomes show just a limited proportion of patients receive benefits from immunotherapy (12). This implies that how to regulate the communication between immune cells and tumor cells based on the complex TME to enhance the antitumor immune response and how to adopt better immunotherapy strategies to ameliorate the clinical survival of cancer patients still need to be explored at present.

Recently, the anti-cancer and immunomodulatory effects of many natural compounds have attracted increasing attention. Tea, as one of the oldest and most prevalent beverages globally, provides many bioactive ingredients, such as theanine, caffeine, vitamins, and various types of catechins that are proven to benefit human health (13, 14). In addition, the scientific community currently generally agrees drinking tea is fairly appropriate for humans, as there are few reports of serious adverse events from drinking tea (15). Generally, according to the difference in the degree of fermentation, tea can be classified into 6 categories: green, white, yellow, oolong, black, and dark teas, among which, green tea is made from the fresh leaves of tea bushes through the process of blanching, rolling, and drying, without specific fermentation, so a large number of catechins are retained (15, 16). Catechins are the foremost polyphenolic substances in green tea, mainly including epigallocatechin gallate (EGCG), epigallocatechin, epicatechin gallate, gallocatechin gallate, epicatechin, and gallocatechin, among which EGCG is the most abundant catechin in green tea (13, 16).

Accumulating evidence supports the antitumor activity of EGCG and reveals its various molecular targets and mechanisms in inhibiting tumorigenesis and progression, involving the modulation of multiple biological behaviors, including tumor proliferation, invasion, migration, apoptosis, autophagy, epigenetic modification, and therapeutic resistance (17–21). For example, in solid tumors, EGCG has been found to prevent tumor growth and proliferation by reducing the activation of molecules such as mitogen-activated protein kinases (MAPK), extracellular signal-regulated kinase (ERK), and protein kinase B (Akt), to induce cell cycle arrest by upregulating p21 transcription, to trigger mitochondrial cytochrome C-mediated apoptosis by diminishing Bcl-2 and Bcl-xL expression, and to attenuate malignant migration and invasion by reducing the levels of matrix metalloproteinases (MMPs) (16, 17, 19). Additionally, EGCG can regulate DNA methylation and histone acetylation levels through interacting with DNA methyltransferases and histone deacetylases to epigenetically control tumor genome expression (22). Furthermore, evidence has revealed that EGCG remarkably overcomes chemotherapy resistance and re-sensitizes tumor cells to multiple chemotherapy drugs, such as cisplatin, 5-fluorouracil, doxorubicin, and daunorubicin (16, 23). Nevertheless, its regulatory roles and relevant mechanisms in TME, metabolic reprogramming, and cancer immunotherapy remain obscure and need to be further elucidated and discussed.

In this review, we summarized the most recent updates about the effects of EGCG on the immune microenvironment, metabolic reprogramming, and immunotherapy. Specifically, we elaborated on the roles of EGCG in the functions of immune cells and stromal cells, and the suppression of EGCG in glucose uptake, aerobic glycolysis, glutamine catabolism, fatty acid anabolism, and nucleotide synthesis in TME, as well as the potential value of EGCG-involved immunotherapy strategies, to provide important references to its clinical application.

## 2 Immunomodulatory roles in immune cells in TME

**Figure 1:**
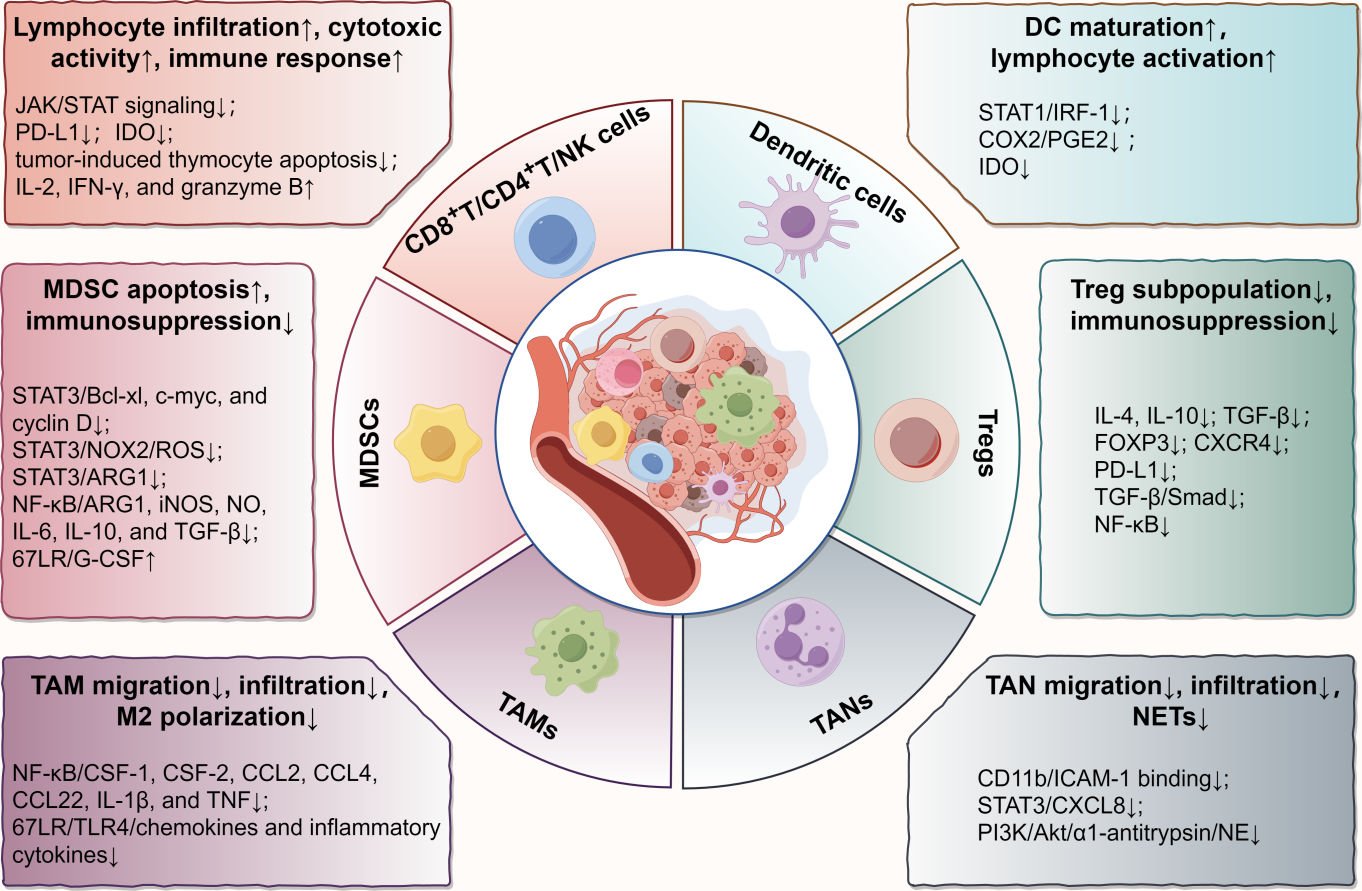
Main effects and molecular mechanisms of EGCG on immune cells, including T lymphocytes, NK cells, DCs, MDSCs, Tregs, TAMs, and TANs in tumor microenvironment. EGCG enhances the infiltration and antitumor immune response of cytotoxic lymphocytes, promotes the maturation and tumor antigen loading of DCs, attenuates the immunosuppressive effect of MDSCs and Tregs, and blocks the regulation of TAMs and TANs on tumor promotion, involving various biomolecular targets, such as STAT, NF-κB, TGF-β, TLR4, PI3K/Akt, PD-L1, IDO, and their relevant signaling pathways. The figure was drawn using Figdraw.

Recently, EGCG has gained a reputation for its immunomodulatory effects in the TME (24). To elucidate how EGCG acts on the immune microenvironment, we reviewed its immunoregulatory roles and relevant molecular targets in various immune cells in TME, including CD8^+^/CD4^+^ T cells, natural killer (NK) cells, dendritic cells (DCs), myeloid-derived suppressor cells (MDSCs), regulatory T cells (Tregs), tumor-associated macrophages (TAMs), and tumor-associated neutrophils (TANs) (**Figure 1**).

### 2.1 CD8^+^/CD4^+^ T lymphocytes

CD8^+^ T cells, commonly known as cytotoxic T lymphocytes (CTLs), are the pivotal effector cells mediating anti-cancer immunity in the tumor immune microenvironment (TIME) (25). When the CTLs recognize the tumor antigens and are activated, they will release perforin and granzyme and initiate Fas ligand-mediated apoptosis to eradicate tumor cells (26). In addition, activated CD4^+^ T cells can not only play an auxiliary role in enhancing the clonal expansion and antitumor activity of CTLs but kill tumor cells through direct cytotoxic effects (27, 28). However, these tumor clearance processes are not so smooth, hampered by various complicated factors in TIME. Thus, how to effectively stimulate or enhance the antitumor immune response of T lymphocytes has always been an important challenge in tumor immunotherapy.

Numerous biological experiments have provided convincing arguments in favor of EGCG as an immunological regulator of T lymphocytes. In the EGCG-treated murine metastatic melanoma cells, upregulated expression of granzyme B in CD8^+^ T lymphocytes was detected in TME (29). In the E7-expressing murine xenograft model, EGCG induced the generation of special CD8^+^ T lymphocytes and enhanced the immune response of these cells (30). For the ultraviolet ray (UV)-exposed mice, EGCG prevented skin tumor development through enhancing the infiltration or recruitment of CD8^+^ T lymphocytes into the TIME (31). Similarly, in a murine WEHI-3 leukemia model, EGCG exhibited an anti-cancer effect through boosting the proliferation of T cells and phagocytosis of macrophages (32). Furthermore, evidence suggested EGCG not only elevated the proportion of CD8^+^ T lymphocytes *in vitro* but significantly increased the CD4 surface intensity on activated T lymphocytes and promoted the Th1 response when combined with resveratrol (33). Even 5-(3’,5’-dihydroxyphenyl)-γ-valerolactone (EGC-M5), a primary metabolic product of EGCG metabolized by intestinal bacteria, was also found to dramatically boost the activity of CD4^+^ T cells and the production of interferon-gamma (IFN-γ) (34). Collectively, cumulative evidence confirmed EGCG plays crucial immunomodulatory roles in the infiltration and activation of T lymphocytes *in vitro* and *in vivo*, which is beneficial for improving the anti-cancer immune response.

The mechanism behind the aforementioned immunomodulatory potential of EGCG for cytotoxic immune cells may be mainly attributed to the following points. Firstly, scientific studies demonstrated EGCG downregulates the expression of programmed cell death ligand 1 (PD-L1) through disrupting the Janus kinase (JAK)/signal transducer and activator of transcription (STAT) signaling pathway in melanoma (29). Due to the removal of PD-L1-mediated immunoinhibitory signals between tumor cells and CTLs in TME, these CTLs are reactivated to resume the antitumor immune response. Secondly, indoleamine 2,3-dioxygenase (IDO), a promoter of immune escape and immune tolerance in TME, can catalyze the catabolism of tryptophan to inhibit the proliferation of T lymphocytes, induce the apoptosis of T lymphocytes, and increase the infiltration of immunosuppressive cells, thus inhibiting the antitumor response of cytotoxic lymphocytes (35–37). With the JAK/STAT signal blocked by EGCG, its downstream IDO expression is subsequently suppressed (35, 38). Thirdly, substantial evidence confirmed EGCG activates the immune response of T lymphocytes by attenuating MDSC-mediated immunosuppression (39, 40). Finally, drawing from previous research, we hypothesized that EGCG might protect the thymus from tumor-induced thymocyte apoptosis and involution by reducing oxidative stress in thymocytes or modulating the JAK/STAT signaling pathway, thereby providing a prerequisite for T cell maturation, proliferation, and differentiation (41, 42). But further investigation is needed to confirm that.

### 2.2 Natural killer cells

NK cells, named for their capacity to automatically kill target cells, have been identified as the principal effector cells with cytotoxicity in the antitumor innate immunity (43). Interestingly, NK cells are equipped with various activating and inhibitory surface receptors that transmit either activating or suppressive signals to regulate their activation (44). For instance, as the most potent activating receptor, Fcγ receptor III also known as CD16, stimulates NK cells through the antibody-dependent cell-mediated cytotoxicity (ADCC) process, which is a crucial antitumor immune mechanism (45). Additionally, in normal cells, highly expressed major histocompatibility complex (MHC) class I (MHC-I) molecules interact with the inhibitory receptors of NK cells to protect themselves from attack by NK cells, while MHC-I molecules are often low expressed in tumor cells to evade CD8^+^ T lymphocyte surveillance (46). Due to the absence of MHC-I-mediated inhibitory signals, NK cells identify this missing-self phenotype and respond to these tumor cells, eventually causing their lysis (44, 45).

In addition to increasing the antitumor immune response of T lymphocytes in the TIME, EGCG has been demonstrated to augment anti-cancer cytotoxicity of NK cells (47). Previous evidence revealed the NK cells extracted from the spleens of the EGCG-administered murine WEHI-3 leukemia model showed significantly enhanced cytotoxic activity against NK-sensitive target cells (murine lymphoma YAC-1 cells) compared to control (32). Soon after, another scientific study detected similar results and came to a consistent conclusion (48). Likewise, in the mouse models with bladder cancer, both EGCG and EGCG nanoparticles enhanced NK cell-mediated cytotoxicity, which appeared to be relevant to significantly upregulated levels of interleukin (IL)-2 and IFN-γ (49). Furthermore, evidence unveiled EGC-M5, a metabolic derivative of EGCG, also upregulated the cytotoxicity of NK cells, which was attributed to the increased levels of granzyme B in NK cells, while EGC-M5 did not affect the populations of NK cells with perforin (34). These findings fully demonstrated EGCG can effectively heighten the antitumor response of NK cells. Nevertheless, the detailed biomolecular mechanisms behind how EGCG regulates NK cell activity remain obscure, and require to be further investigated in the future.

### 2.3 Dendritic cells

As the strongest specialized antigen-presenting cells (APCs) in TIME to present cancer-associated antigens, DCs initiate the adaptive immune response of naive T lymphocytes by upregulating specific MHC and costimulatory molecules after maturation and activation (50). Since DCs regulate the critical immune response in the TME, DC vaccines used to activate antitumor CTLs may be a novel immunotherapy option (51, 52). Meanwhile, how to enhance the function of DCs in antitumor therapy has always been the fundamental area that researchers pay attention to.

Evidence suggested EGCG antagonized UV-induced inhibition of DC function though possibly protecting the levels of costimulatory molecules on the DC surface and regulating the release of certain cytokines, including IL-10 and IL-12 (53). EGCG was also discovered to avoid UV-induced loss of APC population in mouse models, which was possibly related to EGCG alleviating oxidative damage in the dermis as well as the epidermis at UV-exposed sites (54). These findings imply that EGCG may minimize UV-induced immunosuppression against cutaneous melanoma or basal cell carcinoma. Furthermore, some researchers detected more antigen-loaded DCs in the regional lymph nodes of mice with oral EGCG, which were responsible for activating CD8^+^ T cells (30). Notably, EGCG can significantly suppress STAT1 activation and binding to interferon regulatory factor 1 (IRF-1) promoter in IFN-γ-stimulated DCs, and prominently reduce cyclooxygenase-2 (COX-2) expression and prostaglandin E2 (PGE2) generation, thus attenuating the levels of IDO and reversing the IDO-mediated T lymphocyte suppression (38). The downregulation of IDO seems to explain the mechanism by which EGCG affects antitumor immunity through regulating DCs. In terms of studies involving tumor vaccines, evidence suggested that EGCG, as a DC maturation agent, effectively promoted DC maturation and enhanced the anti-cancer effects when administered in combination with mesothelin-specific DNA vaccine, implying this combination application may be a potent immunotherapy strategy against mesothelin-expressing ovarian cancer, pancreatic cancer, and malignant mesothelioma (55). Briefly, EGCG plays a significant regulatory role in DC maturation and immune function, which influences subsequent cytotoxic lymphocyte responses.

### 2.4 Myeloid-derived suppressor cells

MDSCs, a heterogeneous population of immature myeloid cells, belong to pathologically activated monocytes and granulocytes, including monocytic (M-MDSC) and granulocytic/polymorphonuclear (PMN-MDSC) subsets, and exhibit significant immunosuppressive activity in TME (56, 57). Ample evidence has confirmed the immunosuppressive mechanism of MDSCs on CTLs mainly involves the upregulated levels of arginase I (ARG1), inducible nitric oxide synthase (iNOS), and reactive oxygen species (ROS) (58–60). For instance, MDSCs can secrete ARG1 to cause local deprivation of L-arginine in the TME, which will impede CTL activation and inhibit T cell proliferation (60, 61). iNOS can compete with ARG1 for the identical substrate and metabolize L-arginine to produce nitric oxide (NO), which will block the IL-2 receptor signaling and nitrate the T cell receptor to result in immunosuppression (60, 62, 63). Moreover, in addition to mitochondria-generated and tumor-derived ROS, MDSCs produce a great deal of ROS in the TME through NADPH oxidase 2 (NOX2), which reduces CD3ζ expression in T lymphocytes, thereby limiting T lymphocyte activation and corresponding IFN-γ expression (59).

In a murine breast cancer model, several researchers found EGCG ameliorated MDSCs-mediated immunosuppression though reducing the frequencies of MDSCs in the peripheral blood, spleens, and tumor tissues, and *in vitro* experiments also confirmed EGCG prevented the growth and promoted the apoptosis of MDSCs (40). Another highly insightful investigation pointed the mice drinking EGCG-rich polyphenol E (PE) were manifested as a significant reduction in the tumor-infiltrating myeloid cells with the myeloid hallmarks CD11b or Gr-1 and an antagonism to the cancer-promoting effects of tumor-induced myeloid cells on neuroblastoma (39). Interestingly, when the researchers injected neuroblastoma cells into immunodeficient mice that drank PE-containing water, tumor growth was not affected, while the opposite was observed in immunocompetent mice inoculated with neuroblastoma cells (64). Subsequently, increased T-cell infiltration was detected in the murine tumors co-injected with PE-treated MDSCs, and increased tumor volumes were observed as the immune depletion of CD8^+^ T cells (39). These results imply EGCG may directly target MDSCs to suppress their immunoinhibitory activities, thereby playing an antitumor role through functional cellular immunity dependent on CD8^+^ T cells.

Further investigation revealed the inhibitory mechanisms of EGCG on MDSCs were attributed to the regulation of a repertoire of canonical and non-canonical pathways, the former mainly including the STAT3 and nuclear factor-kappaB (NF-κB) signaling pathways, and the latter mainly including the ECM-receptor interaction and focal adhesion (40). Among them, STAT3, the first identified transcription factor relevant to MDSCs expansion in tumors, can block the normal differentiation of myeloid cells and upregulate the expression of multiple antiapoptotic genes, such as Bcl-xL, c-myc, and cyclin D (60, 65). Relevant experiments confirmed activated STAT3 upregulated ARG1 expression in MDSCs through binding to ARG1 promoter, which explains why inhibiting STAT3 can significantly reduce ARG1 and abolish the inhibitory activity of MDSCs (66). Activated STAT3 was also found to induce ROS production in MDSCs through upregulating several subunits of NOX2 to increase ROS-mediated immunosuppression (67). Furthermore, STAT3 can regulate the levels of multiple immunosuppressive facilitators, such as IL-10, PD-L1, and S100 proteins, to promote the proportion and activation of MDSCs (68). Expectedly, evidence suggested EGCG dramatically attenuated STAT3 phosphorylation and downregulated the expression of the aforementioned downstream molecules, possibly due to a direct physical interaction between EGCG and the phosphopeptide binding domain formed by the STAT3 SH2 fold (40, 69). Of note, the phenolic hydroxyl groups of EGCG have strong antioxidant activity based on electron delocalization, which means EGCG can also directly quench the ROS generated by MDSCs in the TME (20). Similarly, EGCG can suppress NF-κB dose-dependently in MDSCs, thereby diminishing a range of downstream immunosuppressive mediators, such as ARG1, iNOS, NO, IL-6, IL-10, and transforming growth factor-beta (TGF-β) (40). Computational docking analysis also confirmed EGCG can directly inhibit NF-κB-mediated transcriptional activation due to the covalent connections between EGCG and p65 subunit (16, 70). Interestingly, EGCG appears to activate the 67 kDa laminin receptor (67LR) and granulocyte colony-stimulating factor (G-CSF) secretion to induce phenotypic differentiation of MDSCs from immature myeloid cells to more mature neutrophil-like cells that have hypersegmented nuclei and fail to inhibit IFN-γ release from CD3 splenocytes (39, 64, 71). In short, these results illuminate the underlying mechanisms by which EGCG targets MDSCs to rescue antitumor immunity.

### 2.5 Regulatory T cells

Tregs are immunosuppressive T cells with the characteristic forkhead box protein 3 (FOXP3) expression, mainly including natural Tregs (nTregs) and induced Tregs (iTregs) (72). Normally, nTregs maintain autoimmune tolerance through attenuating T lymphocyte expansion and cytokine production to prevent an autoimmune response (73). However, in reaction to the stimulation of tumor antigens, TGF-β, and other soluble molecules in TME, peripheral naive T cells enter the TME and transform into iTregs, which together with nTregs recruited into tumor tissues constitute a tumor-associated Tregs pool (74). These Tregs overexpress inhibitory receptors, secrete suppressive cytokines, and disrupt metabolism to create obstacles for immune infiltration of effector cells into TME, which are linked to the poor prognosis of tumors notably (75). Therefore, depleting Tregs is a crucial strategy for enhancing antitumor immunity.

A previous clinical trial reported that in the vast majority of patients suffering from chronic lymphocytic leukemia, oral administration of green tea extracts significantly diminished the absolute number of circulatory Tregs, as well as the serum levels of inhibitory cytokines associated with Tregs (including IL-10 and TGF-β) (76). Another latest clinical trial revealed that in acute myeloid leukemia individuals who received oral green tea extracts (15% EGCG) alone or in combination with cytarabine for at least six months, not only a reduction in the frequencies of Tregs and CXC chemokine receptor (CXCR) 4 Treg subpopulation and the levels of IL-4 and TGF-β were observed, but the activated and cytotoxic phenotypes of CD8^+^ T lymphocytes and NK cells were detected (77). In a co-culture environment involving cancer stem cells and peripheral blood cells from patients with renal clear cell carcinoma, EGCG combined with the first-line treatment sunitinib prominently also decreased the frequency of FOXP3 Tregs in comparison to monotherapy (78). In a murine melanoma model, changes in TME after treatment with EGCG or its nanoparticles were analyzed. The results demonstrated that EGCG or its nanoparticles could significantly inhibit PD-L1 expression and diminish the infiltration of immunosuppressive Tregs, which would ameliorate immune exhaustion and restore the tumor-killing ability of CTLs, thus enhancing the desired antitumor therapeutic effects (79). These suggest that in addition to MDSCs, EGCG may target Tregs to attenuate their immunosuppression in human tumors. Additionally, EGCG may be applied as an immunomodulatory adjuvant in combination with chemotherapy or targeted therapy for tumors. Unfortunately, the available evidence does not directly elucidate the mechanism by which EGCG regulates Tregs. Since TGF-β produced by tumor tissues facilitates the transformation of immature CD4^+^ T lymphocytes into FOXP3 Tregs in TME and activates NF-κB and Smad signaling pathways to induce FOXP3 expression, we highly hypothesize EGCG reduces the number of Treg subpopulation through downregulating TGF-β/Smad expression and blocking NF-κB activity based on the previous relevant research (70, 80–82).

### 2.6 Tumor-associated macrophages

As the critical members of immune cells in TME, TAMs are mainly split up into two subpopulations of immune cells with diametrical functions, namely antitumor M1 macrophages that mediate cytotoxicity and ADCC to eradicate cancer cells and alternatively tumor-promoting M2 macrophages that demonstrably inhibit antitumor cellular immunity, promote neovascularization, and cause tumor progression (83). TAMs can induce the generation of a repertoire of mediators, including various growth factors, cytokines, and chemokines, multiple anti-apoptotic factors mediated by NF-κB, and abundant soluble immunosuppressive factors involved in IL−10, TGF-β, ARG1, IDO, and PD-L1, to reshape the TIME in favor of tumor progression and subvert local immune surveillance (84). Collectively, tumor-infiltrating TAMs in TME often act as both “tumor promoters” and “immunosuppressors”, suggesting that the macrophage-centric approaches are the key strategy for cancer prevention and therapy.

In TME, chemokines are essential for the recruitment and activation of macrophages. It was discovered that EGCG suppressed the migration and infiltration of TAMs through downregulating certain chemokines in TME. A scientific study in murine breast cancer model reported the suppressive effect of EGCG on tumor growth seemed to be closely correlated with the reduced infiltration of TAMs with the downregulation of monocyte chemokines, including macrophage-colony stimulating factor (CSF) -1 and C-C chemokine ligand (CCL) 2 (85). Investigation in endometrial cancer supported that EGCG inhibited the secretion of CXC chemokine ligand (CXCL) 12 from stromal cells, thereby limiting macrophage migration, infiltration, and differentiation toward TAMs (86). Likewise, the transcriptional crosstalk analysis in macrophage-like differentiated leukemia cells revealed EGCG significantly decreased the expression of CCL2, CCL4, CCL22, CSF-1, CSF-2, IL-1β, and tumor necrosis factor (87). Doubtlessly, the downregulation of these chemokines or cytokines is largely related to EGCG-mediated suppression of NF-κB signaling (88–90). Additional evidence unveiled EGCG attenuated activated macrophage migration dose-dependently through inducing the internalization of 67LR, a crucial molecule in cell activation and migration, on the membrane surface of macrophages (91). In fact, EGCG suppresses the toll-like receptor (TLR) 4 signaling via 67LR, thus attenuating the levels of macrophage chemokines and inflammatory cytokines (92, 93). These findings explain the non-negligible inhibitory roles of EGCG in TAMs, which may be closely related to its anti-inflammatory properties.

Besides macrophage recruitment and infiltration, the phenotype of TAMs in TME is also controlled by EGCG. M2 polarization is a common hallmark of TME, with downregulated IL-12 and upregulated IL-10, IL-4, and IL-13 as secretion profiles (94). Scientific evidence in breast cancer showed EGCG-induced upregulation of miR-16 that was transported to TAMs via exosomes and contributed to the inhibition of NF-κB through the downregulation of IKKα and the subsequent accumulation of Iκ-B, thereby resulting in a tilt of TAMs cytokine profiles from M2- into M1-like phenotype and inhibition of M2 polarization (85). This finding provides a novel mechanism for how EGCG exerts antitumor effects through manipulating TAMs in TME. Furthermore, a synergistic combination containing EGCG, curcumin, and resveratrol was reported to promote the phenotypic transformation of M2 macrophages into M1 macrophages through reducing the expression of IL-10 by 70% in the TAMs of murine xenograft tumors, while increasing the expression of IL-12 by 244%, which was considered to be related to suppressed STAT3 expression and inhibited STAT3 phosphorylation (95). Meanwhile, the liposome particles composed of the same formulation were demonstrated to cause M2 TAMs to repolarize into tumor-killing M1-like phenotypes and recruit tumoricidal NK cells in glioblastoma (96). These results provide credence that EGCG may evoke the switch of M2 macrophages to M1 macrophages.

### 2.7 Tumor-associated neutrophils

Recently, the complicated effects of neutrophils on carcinogenesis and progression have received considerable attention. Similar to TAMs, TANs can also be categorized into anti-cancer phenotype, which kills cancer cells by direct cytotoxic actions and indirect activation of adaptive immune responses, and alternatively cancer-promoting phenotype, which is involved in tumor proliferation, angiogenesis, and immunosuppression (97, 98). Evidence has demonstrated TANs not only promote invasion and angiogenesis by producing MMP-9, vascular endothelial growth factor (VEGF), and hepatocyte growth factor (HGF) but also promote distant migration and dissemination through ensnaring tumor cells via neutrophil extracellular traps (NETs) (99). Sufficient studies have also confirmed a high neutrophil-to-lymphocyte ratio is indeed linked to an unfavorable prognosis in multiple cancers (100–102). Hence, drugs targeting TANs offer a bright future for antitumor immunotherapy.

As early as the 1990s, researchers began to explore the roles of EGCG in neutrophil migration and infiltration. Wei et al. discovered EGCG attenuated the infiltration of neutrophils caused by phorbol ester-type skin tumor promoters in the skin tissue of SENCAR mice, and attributed this phenomenon to the inhibitory effect of EGCG on oxidative events (103). Hofbauer et al. mimicked neutrophil transmigration from the vascular lumen to tissue and found EGCG significantly suppressed the migration of neutrophils through monolayer endothelial cells after treating both of them with relevant plasma concentrations of EGCG (104). Such a finding suggests that EGCG may interfere with the entry of circulating neutrophils into TME. Subsequently, EGCG was found to inhibit neutrophil infiltration by acting directly on neutrophils, independent of both the cytotoxicity of EGCG and the influence of chemical inducers (105). However, the possible mechanisms behind this were not revealed, until one scholar explained this inhibitory effect of EGCG on neutrophils depends on the strong interactions between EGCG and CD11b expressed on the neutrophil surface, which competitively suppresses the binding of specific molecules to the CD11b and particularly leads to a markedly reduced ability of neutrophils to bind intercellular adhesion molecule 1 (ICAM-1), an adhesion molecule mediating the passage of neutrophils through endothelial cells (106). Additionally, a recent report illuminated EGCG blocked NET formation as well as STAT3 and CXCL8 expression in the colon cancer-derived TANs, thereby inhibiting the invasion and migration of colon tumor (107). Briefly, the blocking of CD11b/ICAM-1 signal and inhibition of STAT3/CXCL8 pathway may be the key mechanisms by which EGCG suppresses neutrophil migration and infiltration into the TME.

Neutrophil elastase (NE), a major serine protease of neutrophils, is stored in the primary granules of neutrophils and released into the surrounding environment with neutrophil stimulation and activation (108). Recently, scientists have come to realize NE is likely to be a driver of tumorigenesis, as NE significantly promotes tumor growth by reshaping TME, and NE suppression observably diminishes tumor burden and metastasis (109, 110). EGCG directly binds to NE via multiple hydrogen bonds and subsequently suppresses these enzyme activities, and increases the levels of α1-antitrypsin, a natural inhibitor of NE, by weakening the phosphorylation of phosphatidylinositol 3-kinase (PI3K)/Akt pathway, thereby reversing NE-induced migration of tumor cells (111). Furthermore, the inhibitory functions of EGCG in NE activity were also confirmed by biochemical assays, with the 50% inhibitory concentration ranging from 10.9μM to 25.3μM (112–114). Therefore, there is substantial evidence that EGCG can inhibit the enzyme activity of NE released by activated TANs in TME to prevent tumor progression.

## 3 Regulatory effects on stroma cells in TME

**Figure 2:**
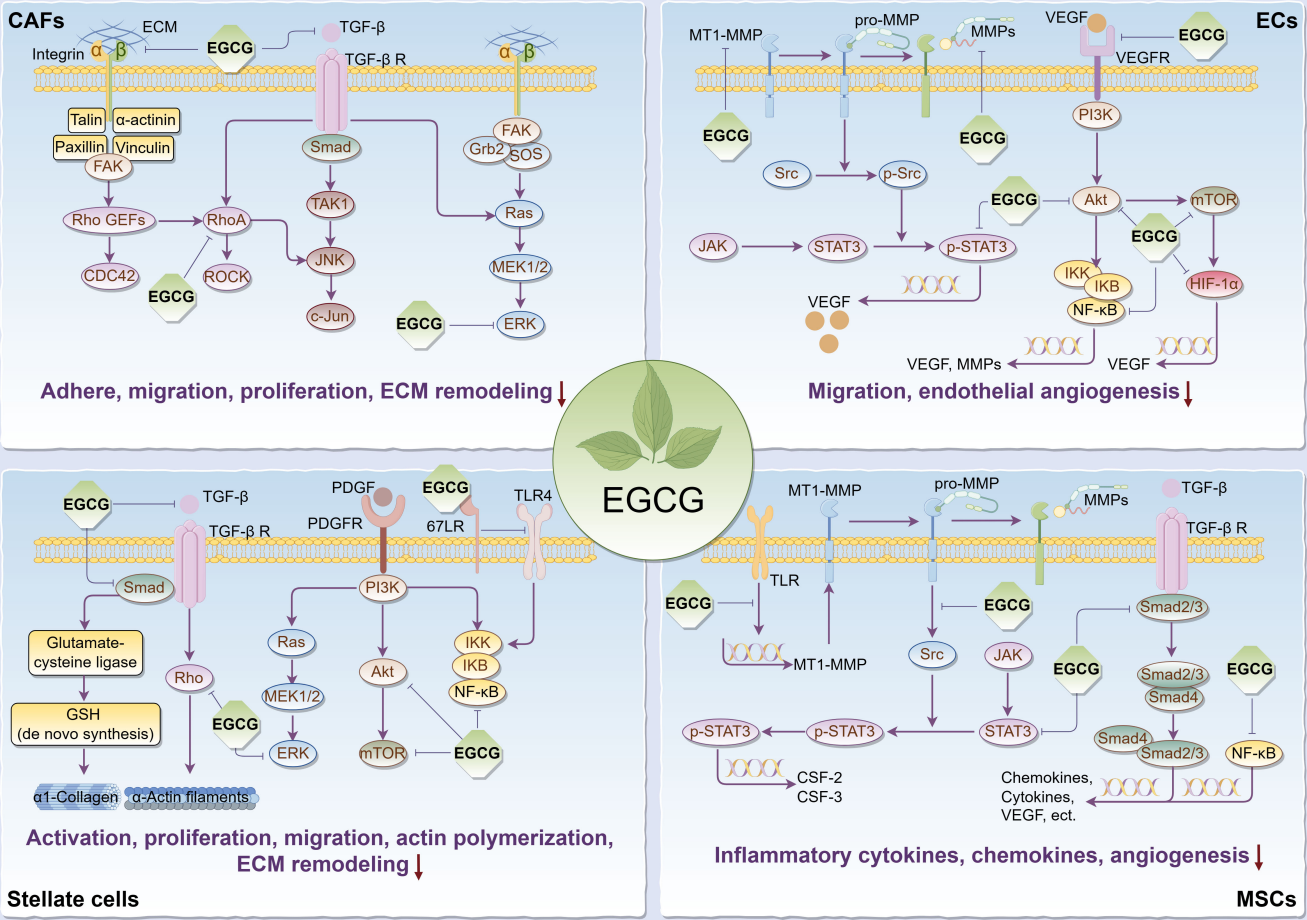
Main effects and molecular mechanisms of EGCG on tumor stromal cells, including CAFs, ECs, stellate cells, and MSCs in tumor microenvironment. EGCG inhibits tumor stromal cell activation, adhesion, proliferation, migration, inflammatory cytokine and chemokine secretion, actin polymerization, ECM remodeling, and angiogenesis. Main molecular mechanisms may be attributed to the inhibitory effect of EGCG on Rho/ROCK, ECM/integrin, RTKs/PI3K/Akt/mTOR, TGF-β/Samd, MAPK/ERK, JAK/STAT3, NF-κB, 67LR/TLR4, and HIF-1α signaling pathways. The figure was drawn using Figdraw.

It’s commonly known that tumorigenesis is usually accompanied by the formation of the tumor bed as well as the remodeling of the surrounding extracellular matrix (ECM), in which tumor stromal cells play a pivotal role, thus forming a suitable TME for tumor cell survival, so targeting relevant stromal cells is critical to cancer treatment. Given that, we reviewed the regulatory effects and relevant biomolecular mechanisms of EGCG on stroma cells in TME, including cancer-associated fibroblasts (CAFs), endothelial cells (ECs), stellate cells, and mesenchymal stem/stromal cells (MSCs) (**Figure 2**).

### 3.1 Cancer-associated fibroblasts

CAFs, the highly heterogeneous stromal cells in TME, play versatile roles in ECM remodeling, inducing angiogenesis, modulating tumor metabolism, excluding immune response, and promoting tumor progression and therapeutic resistance based on intricate signaling crosstalk with tumor cells (115, 116). However, CAF-centered clinical trials have mostly ended in failure, which means the therapeutic strategies by modulating CAFs to achieve clinical benefits face many challenges (117, 118). Fortunately, EGCG offers promising prospects for improving the TME by targeting CAFs.

As the “bad neighbors” of tumor cells, the proliferation and migration of CAFs are considered necessary for tumor progression. According to one study, EGCG prevented fibroblast adhesion to various ECM proteins by possibly binding to fibronectin and fibrinogen, affecting the expression and affinity of integrin α2β1, and reducing the phosphorylation of focal adhesion kinase (FAK) and the organization of actin cytoskeleton (119). Simultaneously, EGCG reduced fibroblast migration on matrix proteins, which appears to be attributable to its suppressive effects on the expression and activity of MMPs in the stromal fibroblasts (119, 120). Some scholars found that in keloid, a benign skin tumor, EGCG significantly suppressed the proliferation, migration, and collagen production of pathological fibroblasts through blocking the STAT3 signaling, and this inhibitory effect on proliferation and migration was stronger than that of normal fibroblasts (121). These observations largely demonstrate EGCG is a potent inhibitor of stromal fibroblast behaviors. Nevertheless, whether EGCG induces fibroblast apoptosis remains controversial. Hung et al. considered the inhibitory roles of EGCG in fibroblasts were independent of its cytotoxic or pro-apoptotic activity, as the same concentration of EGCG had a specific suppressive effect on fibroblast adhesion to different matrix proteins, and no increase in lactate dehydrogenase (LDH) release and decrease in cell viability were detected during fibroblast culture treated with EGCG (119). Conversely, in the abnormal fibroblasts from transgenic mice overexpressing tumor-associated NADH oxidase, low concentrations of EGCG inhibited their growth, and their apoptosis was observed 48 hours later (122). This difference may be related to different sources of fibroblasts or different experimental conditions. Thus, further *in vitro* and *in vivo* experiments are urgently needed to verify EGCG’s roles in the apoptosis of CAFs.

Not only that, there is additional evidence to support EGCG can impede cancer progression by targeting CAFs. In thioacetamide-induced rat hepatoma assay, EGCG effectively diminished hepatic fibrosis and fibroblast growth factor that can stimulate angiogenesis and proliferation of fibroblasts (123). A phase II clinical trial explored the effect of EGCG supplementation on biological markers in patients with prostate carcinoma before surgery, with the results suggesting a substantial reduction in the tumor-promoting HGF and VEGF levels (124). Further *in vitro* investigations confirmed the potential of EGCG to suppress the production of these two biomarkers in prostate CAFs. Considering that these two notorious biomarkers are primarily secreted from tumor myofibroblasts in TME, that implies EGCG may inhibit the differentiation of CAFs into prostate cancer-associated myofibroblasts. Then the researchers unveiled EGCG combined with luteolin in prostate CAFs synergically inhibited TGF-β-mediated myofibroblast phenotypes with fibronectin expression and diminished TGF-β-mediated ECM contraction, a promoter of tumor invasion (125). Mechanistically, both EGCG and luteolin attenuated the signaling activation of Ras homolog family member A (RhoA) necessary for TGF-β-mediated fibronectin expression in CAFs and inhibited downstream TGF-β-induced signaling, such as ERK (125, 126). Meanwhile, as a potential inhibitor of RhoA, EGCG may also inhibit the recruitment and persistent education of CAFs in breast cancer by blocking the RhoA/Rho-associated kinase (ROCK) signaling pathway, thus suppressing the invasion of tumor cells (127, 128). In addition, a recent study revealed EGCG obstructed aerobic glycolysis in CAFs to disrupt their tumor-promoting capabilities. Taken together, EGCG can target CAFs to reverse tumor progression.

### 3.2 Endothelial cells

Among the tumor stroma cells, ECs are the primary contributors to the formation of the tumor vascular system. Even more to the point, tumor-associated endothelial cells (TECs) evolve into mesenchymal phenotypic cells by endothelial-mesenchymal transition, which drives various cancerous biological characteristics of tumors, including aberrant angiogenesis, CAF formation, tumor metabolism, invasion, metastasis, and resistance to treatment (129, 130). Given this, ECs are one of the key targets of antitumor therapy.

The transformation of ECs into capillaries requires endothelial growth, migration, invasion, and matrix remodeling (131). Extensive evidence has confirmed EGCG can suppress these angiogenic features. A previous study reported EGCG could not only inhibit endothelial growth but reduce endothelial migration and capillary tube formation on Matrigel, which was considered to be related to its inhibitory roles in the activities of MMPs during endothelial morphogenesis (132). Soon, several researchers using the dorsal air sac model found EGCG attenuated the invasion and tumor angiogenesis of human ECs, which was attributed to the effective inhibition of membrane-type 1 MMP (MT1-MMP) involved in MMPs activation (133). Subsequent research revealed EGCG dose-dependently reduced the expression and transcriptional activity of MMP-9 in ECs, at least by suppressing ROS, NF-κB, and activating protein-1, thereby blocking the invasion of ECs (134). In addition to EGCG directly targeting TECs, EGCG was found to inhibit VEGF-mediated mobilization of endothelial progenitors into the circulating bloodstream through suppressing MMP-9 generation in marrow stromal cells (135). Notably, a study reported EGCG’s inhibitory effects on the abovementioned angiogenic features of ECs may be related to the inhibition of angiopoietin-2 secretion, which promotes vascular remodeling and sprouting (136). Some scholars, meanwhile, supported EGCG inhibited VEGF-induced migration of TECs through suppressing Akt phosphorylation in the PI3K/Akt signaling pathway during endothelial angiogenesis (135). Contrarily, this anti-angiogenic effect was not observed in normal ECs. Similar evidence also demonstrated EGCG inhibited endothelial morphogenesis through the suppression of VEGF receptor (VEGFR) binding (137). Furthermore, a recent study co-cultured neuroblastoma cells with human ECs in a novel three-dimensional *in vitro* model and found EGCG might inhibit the tumor vascularization microenvironment of TECs (138). These findings imply EGCG can directly block the angiogenic capacity of TECs in TME.

In TME, tumor cells typically produce high levels of VEGF, which is essential to support tumor growth and survival. These proangiogenic VEGF molecules can decisively drive new angiogenesis within tumors through inducing endothelial sprouting, proliferation, and migration and regulating the recruitment of circulating endothelial progenitors (139). Additionally, autocrine VEGF by ECs can also promote endothelial elongation, network formation, and branch (140). Therefore, targeting VEGF/VEGFR signaling has become a major treatment option for many cancers. A compelling body of evidence revealed EGCG can downregulate the expression levels of VEGF by inhibiting multiple tumor-related signaling molecules, such as PI3K, Akt, mammalian target of rapamycin (mTOR), hypoxia-inducible factor-1alpha (HIF-1α), NF-κB, and STAT3, thus inhibiting endothelial angiogenesis and tumor progression (16, 86, 141–143). Furthermore, studies confirmed EGCG significantly reduced basic fibroblast growth factor (bFGF) levels in ECs and tumor cells, by increasing bFGF ubiquitination and 20S proteasome activity, which led to bFGF degradation (144, 145). This is another pathway by which EGCG inhibits endothelial angiogenesis.

Accumulating evidence suggests EGCG in combination with other flavonoids or angiogenesis inhibitors can effectively inhibit endothelial angiogenesis. EGCG combined with silibinin synergistically facilitated the downregulation of proangiogenic VEGF, VEGFR2, and miR-17-92 family and the upregulation of anti-angiogenic miR-19b, when ECs were cocultured with tumor cells (146). Analogously, in comparison to EGCG alone, combined administration of EGCG and curcumin better attenuated the evolution of normal ECs to TECs stimulated by tumor medium supernatant or tumor tissue homogenate through inhibiting the JAK/STAT3/IL-8 pathway, thereby reversing endothelial angiogenesis (147). Furthermore, EGCG markedly decreased endoglin levels in human ECs treated with semaxanib, a potent and selective inhibitor of VEGFR, thus exerting its anti-angiogenic function through targeting proangiogenic endoglin/Smad family member 1 (Smad1) signaling, which means combination therapies including EGCG will be promising to address the resistance of tumor cells to anti-VEGF therapies (148).

### 3.3 Stellate cells

Stellate cells, resident lipid storage cells in the liver and pancreas, can differentiate into a myofibroblast phenotype in response to tissue injury, participating in pathologically inflammatory processes that result in the fibrosis of tumor tissues and the construction of growth-permitting TME (149). In the liver microenvironment, on the one hand, perivenous hepatic stellate cells (HSCs) are identified as the primary source of cancer-promoting CAFs after chronic liver injury (150). On the other hand, HSCs can generate growth differentiation factor 15 to promote the cell growth of hepatocellular carcinoma (HCC) (151). Additionally, evidence supports stellate cells can communicate with immune cells to promote tumor progression. For instance, the crosstalk between HSCs and TAMs drives the premalignant and malignant hepatic microenvironment through ECM remodeling, immunosuppression, and pro-inflammatory cytokines (152). Even, HSCs inhibit breast cancer dormancy maintained by NK cells, since CXCL12 secreted by activated HSCs can induce NK cell quiescence through its homologous receptor CXCR4, which is closely related to tumor liver metastasis (153).

A compelling body of studies have demonstrated EGCG can restrain stellate cell activation and reverse stellate cell-mediated ECM remodeling. EGCG was found to disrupt TGF-β signaling through downregulating TGF-β receptor and Smad4 expression, resulting in stimulating the expression of glutamate-cysteine ligase to enhance *de novo* glutathione synthesis in HSCs, thereby inhibiting HSC proliferation associated with oxidative stress (154). Additional experiments suggested *de novo* glutathione synthesis was necessary for EGCG to block TGF-β signaling and diminished the expression of α1(I) collagen in activated HSCs (155). Meanwhile, it has been reported that EGCG abolished stress-fiber formation and altered α-actin distribution by inhibiting the Rho signaling required for HSC activation and proliferation (156). Furthermore, previous scientific studies reported EGCG blocked the proliferation and migration of hepatic and pancreatic stellate cells induced by platelet-derived growth factor (PDGF), a potent mitogen for stellate cells and regulator of early proliferative response (157, 158). The mechanisms mainly involve its inhibitory roles in the binding between PDGF and its receptor (PDGFR), the phosphorylation of PDGFR, and the signal transduction of downstream PI3K/Akt, ERK, NF-κB, and transcription factors activator protein-1 (157–159). Moreover, evidence supported EGCG suppressed the activation of HSCs possibly through its inhibitory effect on osteopontin, and hindered collagen fiber deposition in the ECM of hepatocytes, thereby reducing fibrosis in the liver microenvironment (160, 161). A recent report also confirmed EGCG blocked HSC activation and attenuated ECM deposition and fibrosis by inhibiting glutamate dehydrogenase (GDH) in glutamine metabolism pathway (162). Collectively, EGCG effectively prevents stellate cell activation and the resulting ECM remodeling by the above multiple pathways, which provides crucial insights into its antitumor targets.

In HCC, it was previously believed that the recurrence and metastasis after radiotherapy were only attributed to the invasive effects of remaining hepatoma cells, whereas it is now realized that this is closely relevant to the enhanced metastasis potential of remaining hepatoma cells caused by radiation treatment (163). There is evidence to support that radiotherapy-induced activation of HSCs provides favorable conditions for promoting the metastasis potential of HCC (164). *In vitro* experiments confirmed that radiotherapy enhanced TLR4 signaling in HSCs and upregulated ICAM-1, 67LR, IL-6, and CX3C chemokine ligand 1, among which EGCG bound to 67LR to inhibit TLR4 signaling and radiation-induced HSC invasion (163). This means EGCG may be beneficial to ameliorate the radiotherapeutic efficacy of HCC and decrease the chance of recurrence and metastasis after radiotherapy. In addition to attenuating the activation of HSCs, EGCG was found to inhibit HCC development by inducing the senescence of HSCs. Both *in vivo* and *in vitro* experiments detected EGCG triggered a series of senescence phenotypes in activated HSCs (165). However, the mechanism of how EGCG regulates the senescence process of HSCs in HCC remains unclear up to now.

### 3.4 Mesenchymal stem/stromal cells

MSCs, which are found in almost all human tissues and are given the ability to transdifferentiate into various connective tissue lineages, such as adipocytes and chondrocytes, can migrate to the tumorigenic sites to participate in the formation of tumor stroma (166). Substantial experimental data indicate the overall net effect of MSCs is toward promoting tumor progression (167). As a vital component of TME, MSCs facilitate tumor cell survival, support angiogenesis, promote epithelial-mesenchymal transition, and increase immune escape from the immune system (168, 169). As a result, increasing scholars have realized targeting MSCs in the TME may be an underlying strategy to ameliorate tumor patient outcomes.

Relevant data illuminated the regulatory effect of EGCG on the signaling cascade of MSCs, that is, EGCG attenuated TLR signaling and subsequent MT1-MMP expression, causing the suppression of MT1-MMP-mediated sequential phosphorylation of Src, JAK, and STAT3, thereby inhibiting the expression of CSF-2 and CSF-3 responsible for endothelial angiogenesis in TME (170). In triple-negative breast cancer (TNBC), EGCG was found to inhibit the paracrine crosstalk between adipose-derived MSCs and TNBC cells. For instance, on the one hand, evidence suggested EGCG prevented a pro-inflammatory and tumor-associated adipocyte-like phenotype (with upregulated CCL2, CCL5, CXCL8, IL-1β, IL-6, COX2, HIF-1α, and VEGF) induced by TNBC secretome in adipose-derived MSCs, primarily through the inhibitory effects of EGCG on Smad2 and NF-κB signaling pathways (171). On the other hand, an investigation revealed EGCG suppressed the differentiation of MSCs into adipocytes and prevented STAT3-mediated paracrine carcinogenic control of TNBC invasion phenotypes in response to adipocytes secretome (172). Nevertheless, apart from paracrine connections to tumor cells, whether EGCG can reverse MSC-induced changes in the immune microenvironment remains unclear. A host of research needs to be performed before further elaboration.

## 4 Regulatory roles in tumor metabolic reprogramming

**Figure 3:**
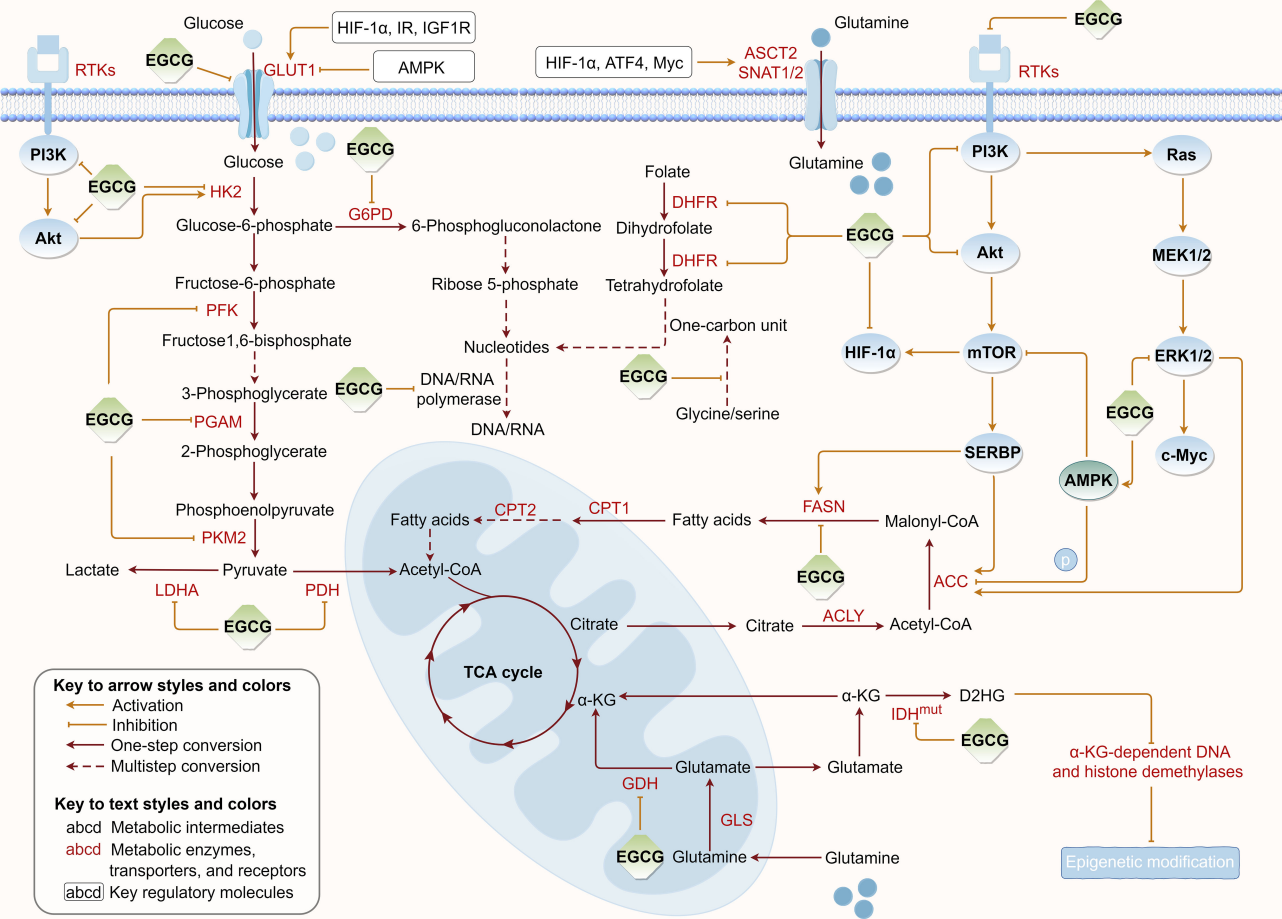
Regulation of EGCG on tumor metabolic reprogramming. EGCG downregulates the expression or/and directly inhibits the activity of various key metabolic enzymes and molecular proteins required for glucose uptake, aerobic glycolysis, glutamine metabolism, fatty acid anabolism, and nucleotide synthesis pathways. The figure was drawn using Figdraw.

Tumor metabolic reprogramming provides a constant supplement to tumor energy consumption and biosynthesis to meet uncontrolled proliferation, which concurrently provides critical targets for tumor therapy. To further elucidate EGCG’s suppressive effects on tumor cell metabolism, we summarized the regulatory roles of EGCG in multiple upregulated metabolic pathways, including glucose uptake, aerobic glycolysis, glutamine metabolism, fatty acid anabolism, and nucleotide synthesis (**Figure 3**).

### 4.1 Glucose uptake

The metabolic flux of glucose depends on glucose uptake by tumor cells, during which glucose enters tumor cells via active transport. On the cell membrane of tumor cells, glucose transporter (GLUT) proteins are often upregulated to promote glucose uptake and support tumor metabolism (173). Upregulated GLUT1 levels have been confirmed to be an important indicator of poor prognosis in multiple cancers, including lung, pancreatic, colorectal, ovarian, breast, bladder, esophageal, and oral cancers (174). Therefore, targeting GLUT offers excellent prospects for antitumor therapy.

A host of experimental findings shed light on the targeted inhibitory roles of EGCG in GLUT-dependent glucose uptake in tumors. Following exposure of colon cancer cells to different EGCG dosages, a sustained reduction in GLUT1 mRNA levels was detected (175). This inhibitory effect appears to be closely correlated with adenosine monophosphate (AMP)-activated protein kinase (AMPK) signaling after EGCG treatment. Similarly, EGCG was observed to reduce GLUT1 expression in breast cancer and pancreatic cancer, although the mechanism may be attributed to its inhibitory effect on HIF-1α, which is vital for regulating the genes encoding GLUT and ameliorating the activities of glycolytic enzymes (176, 177). As far as we know, EGCG’s inhibitory effects on HIF-1α may partially result from the suppression of upstream insulin receptor (IR) and insulin-like growth factor-1 receptor (IGF1R) by EGCG (177). In addition, EGCG was discovered to limit GLUT-mediated glucose uptake through directly and competitively inhibiting GLUT1 in breast cancer cells (178). Relevant molecular docking also supported the direct interaction of EGCG with the glucose docking sites at the core of GLUT1 through hydrogen bonding and hydrophobic contacts (179). Briefly, EGCG can inhibit GLUT1 expression and target GLUT1 activity to interfere with glucose metabolism in tumor cells.

### 4.2 Aerobic glycolysis

Normal cells obtain energy by oxidative phosphorylation, while most solid tumor cells depend mainly on glycolysis to generate energy to adapt to their TME even when oxygen is abundant, a phenomenon named the “Warburg effect” (180). This pattern of energy supply seems uneconomical on the surface, but it is essential for tumor cells because it provides them with a survival advantage and makes their TME more favorable to tumor progression (181). Hence, targeting the “Warburg effect” of tumor cells is an extremely promising treatment approach. This means that in the “Warburg effect”, many crucial regulatory enzymes, including hexokinase (HK), phosphofructokinase (PFK), phosphoglycerate mutase-1 (PGAM1), pyruvate kinase (PK), and LDH can be considered as potential targets for antitumor drug development (182). It has been reported that a novel biological orthogonal probe was used to identify the direct target protein of EGCG based on *in situ* global proteomics profiling, and subsequent gene ontology analysis revealed these main targets belonged to enzymes regulating pivotal metabolic processes such as glycolysis and energy homeostasis (183). This investigation declared a unique EGCG interactome and provided an important reference for EGCG to regulate the metabolic process of glycolysis.

HK can catalyze the phosphorylation of hexose’s sixth hydroxyl group to generate glucose-6-phosphate, the first rate-limiting stage in glycolysis. Preclinical experiments confirmed that EGCG dose-dependently suppressed the activity and mRNA levels of HK2, reduced glucose consumption, and impaired lactate and adenosine triphosphate (ATP) production, and thus the tumor growth was remarkably inhibited (176). Related evidence further pointed EGCG could significantly downregulate HK2 expression through strongly inhibiting epidermal growth factor receptor (EGFR) activation and downstream Akt phosphorylation, thus exerting its far-reaching antitumor effects through direct regulation of glycolysis (184). Molecular docking and dynamics simulation analysis also identified EGCG with obvious binding preference and selectivity for HK2 binding pocket, and could preferentially attach to the corresponding active site to form stable protein-ligand complexes (185). Taken together, these results imply EGCG, as a potential HK2 inhibitor, can potently inhibit the initiation process of glycolysis.

PFK is the second rate-limiting enzyme in glycolysis, responsible for catalyzing fructose-6-phosphate to fructose-1, 6-bisphosphate and mediating glycolytic flux in tumor progression (186). To date, an accumulating body of studies have confirmed the negative regulatory roles of EGCG in the activity and expression level of PFK. Previous investigators, while discovering the inhibitory effects of EGCG on GLUT1 and HK2, also noticed that PFK was an important antitumor target in breast and pancreatic cancer (176, 177). Additional researchers confirmed that EGCG mainly disrupted the glycolysis of pancreatic cancer cells through inhibiting the rate-limiting enzyme PFK, but this inhibitory effect was largely eliminated by catalase, suggesting its inhibitory effects on these enzymes depends on the amount of EGCG-induced hydroperoxide (187). Notably, when EGCG combined with gemcitabine was used to treat pancreatic cancer cells, the researchers detected the activity and protein expression of PKF were further inhibited, and the antitumor effect was further enhanced (187). This means EGCG as a strong combination partner for chemotherapy drugs can sensitize pancreatic cancer cells to gemcitabine. Furthermore, in HCC, to determine whether EGCG directly affects PFK, some researchers analyzed the activity of the purified enzyme. The results clearly indicated EGCG induced PFK transition from the fully active tetramers to the quite inactive dimers, implying its inhibitory role in glycolysis is primarily to reduce PFK activity by stabilizing it in an inactive oligomer conformation (188). Coincidentally, EGCG combined with sorafenib further inhibited PFK expression and tumor cell growth in HCC cells compared with EGCG or sorafenib alone, which provided another valuable reference for antitumor-targeted combination administration (188).

PK is the third crucial rate-limiting enzyme in glycolysis, catalyzing phosphoenolpyruvate to pyruvate. For pancreatic cancer, there has been sufficient evidence to demonstrate EGCG can effectively limit the expression and activity of PK (177, 187). For example, *in vitro* experiments showed 40μM EGCG reduced PKM2 levels in pancreatic cancer cells by 34% to 49%, and *in vivo* experiments demonstrated PKM2 levels were significantly decreased in murine pancreatic tumor xenografts (187). However, in breast cancer, EGCG suppressed the expression and activity of PK to a lesser extent, only reaching significance when treated with 80μM EGCG (176). Conversely, EGCG with the same concentration gradient was indicated to exert its inhibitory effects on HK and PFK to a greater extent. These findings imply that EGCG regulates PK in tumor glycolysis with certain differences, which may be the result of dependence on specific signaling pathways.

In addition to the aforementioned rate-limiting enzymes for glycolysis, evidence indicated EGCG could inhibit other metabolic enzymes associated with the “Warburg effect”. For instance, EGCG significantly inhibits LDH in pancreatic cancer cells, an enzyme that catalyzes the conversion of pyruvate into lactate, and this suppression was observed to be comparable to that of oxamate, an inhibitor of LDH (177, 189). Additionally, screening analyses based on natural compounds identified the inhibitory effect of EGCG on PGAM1 tremendously exceeded that of known PGAM1 inhibitors (190). Further molecular interaction analysis also confirmed EGCG constituted eight hydrogen bonds with the residues of PGAM1, with the main hydrogen bonds from its phenol hydroxyl groups (190). Moreover, evidence revealed EGCG inhibited the expression of pyruvate dehydrogenase E1-α (PDHA1), a key regulator of pyruvate dehydrogenase (PDH), in primary effusion lymphoma, thereby controlling the metabolic flux between glycolysis and the tricarboxylic acid cycle (TCA) (191). Taken together, targeting these vital enzymes associated with glycolysis based on the versatile roles of EGCG is a viable approach for antitumor treatment. Nevertheless, though the growing number of evidence demonstrated a multi-pharmacological mode of action of EGCG on aerobic glycolysis of tumors, the underlying mechanism remains to be further investigated.

### 4.3 Glutamine metabolism

Besides glucose, glutamine is another vital nutrient supporting tumor cell survival, as well as a crucial carbon and nitrogen reservoir necessary for tumor growth and proliferation, supplying ribose, non-essential amino acids, citrate, and glycerol essential for tumor metabolism and supplementing the decreased oxidative phosphorylation caused by “Warburg effect” (192). Evidence has demonstrated tumor cells often overexpress mitochondrial glutaminase (GLS) to catalyze the hydrolysis of glutamine to glutamate that is further converted to α-ketoglutarate (α-KG) by GDH or aminotransferase (162, 193). This means that with the glutamine metabolic pathway rewired in TME, glutamine continuously replenishes the intermediate α-KG for the TCA cycle to support the energy consumption caused by uncontrolled cell proliferation, leading to the consequences of cellular addiction to glutamine (194, 195). The reprogramming limits the glutamine utilization by immune cells and regulates PD-L1 levels of cancer cells, thus affecting the antitumor immune response and aiding tumor immune escape (196).

One study found EGCG effectively reduced total and sodium-dependent glutamine uptake in tumor cells (197). Unfortunately, it is unclear from current studies whether this suppression is related to the expression levels and transport activities of glutamine transporters including alanine/serine/cysteine transporter 2 (ASCT2), and sodium-coupled neutral amino acid transporter 1 and 2 (SNAT1, SNAT2), although these glutamine transporters are often upregulated in tumors. Relevant evidence has revealed the transcriptional programs of these transporters are largely regulated by HIF-1α, ATF4, and Myc (198, 199). Indeed, it has been observed that EGCG can markedly reduce the expression of these transcription factors in tumors (16, 200, 201). Given that, we speculate EGCG may downregulate the expression levels of the abovementioned glutamine receptors, but direct experimental results are still needed to confirm our hypothesis. In addition, to clarify the effect of EGCG on glutamine transporter activities, molecular docking simulations and dynamics analyses should be performed to explore the direct interaction of EGCG with them.

In tumor cells, GDH is the metabolic bridge between glutamate derived from glutamine metabolism and the TCA cycle. Similar to targeting rate-limiting enzymes in glycolysis, targeting GDH also can interfere with the energy generation of tumor cells. There have been an increasing number of studies, including tumor metabolomics analyses, shedding light on the inhibitory roles of EGCG in tumor glutamine metabolism by targeting GDH (202). Previous investigation demonstrated EGCG reduced GDH expression to disrupt glutaminolysis in primary effusion lymphoma cells, and EGCG-induced tumor cell death was attenuated if the deficient α-KG was supplemented (191). Meanwhile, a scientific study suggested EGCG could dose-dependently target GDH activity, which leads to increased oxidative stress and thus makes tumor cells sensitive to radiotherapy (203). Corresponding molecular dynamics simulations supported the abilities of EGCG to establish multiple non-bonding contacts at the adenosine diphosphate (ADP) activation site of GDH and to form multiple hydrogen bonds with a variety of amino acid residues of GDH (204). These results suggest that EGCG can block glutaminolysis for the energy supply of tumor cells.

Notably, isocitrate dehydrogenase (IDH) mutations are often found in multiple tumors, which induce the production of a new pro-oncogenic oncometabolite, D-2-hydroxyglutarate (D2HG), at the cost of α-KG, thus resulting in tumor metabolic alterations (203). Accumulation of D2HG can competitively block α-KG-dependent DNA and histone demethylases, to change epigenetic modifications, thereby indirectly regulating oncogene and anti-oncogene expression (191, 203). In response to IDH^mut^-mediated metabolic stress, tumor cells activate a rescue mechanism composed of the glutamine metabolic pathway, which is accompanied by glutamine and glutamate becoming carbon donors to D2HG (203). Interestingly, there has been a growing body of evidence that EGCG significantly suppresses GDH and IDH activity to reduce D2HG production in tumor cells (191, 203). However, we have to recognize that EGCG is a pleiotropic compound that may have some undiscovered effects besides GDH and IDH inhibition, for instance, whether EGCG can inhibit glutamine metabolic pathway through targeting GLS is currently unknown.

### 4.4 Fatty acid anabolism

Fatty acids, the carboxylic acids with long hydrocarbon chains, are the main components of cellular phospholipids, sphingolipids, glycolipids, and triglycerides, which are mainly obtained from *de novo* lipogenesis (DNL) pathway or directly taken from the extracellular. In the DNL pathway, citrates produced by the TCA cycle are successively catalyzed by ATP citrate lyase (ACLY), acetyl-CoA carboxylase (ACC), and fatty acid synthase (FASN) to produce primary fatty acids (205). They can participate in various metabolic pathways to synthesize more complex lipids, such as diacylglycerols and triacylglycerols, or to convert to phosphatidylglycerols, such as phosphatidylethanolamine and phosphatidylserine (205). Although their biosynthesis in cancer has received relatively little attention compared to aerobic glycolysis and glutaminolysis, it has been recognized in recent years as a significant metabolic aberration necessary for tumorigenesis (206). There is considerable evidence of increased DNL in tumor cells, which may be due to a response to the reduced availability of serum-derived lipids in TME (207). Scientific research supports that the phenotypic reprogramming of fatty acid metabolism is strongly correlated with tumorigenesis, progression, and metastasis and worsens clinical outcomes of tumor patients (208, 209). In addition, this metabolic reprogramming in TME may lead to functional alterations of infiltrated immune cells, thus affecting the efficacy of antitumor immunotherapy (210). Therefore, targeting the DNL pathway in tumors has become an emerging strategy for antitumor therapy and a focus for researchers.

ACC, a rate-limiting enzyme in the reprogrammed DNL pathway, catalyzes the transformation of acetyl-CoA into malonyl-CoA (205). It has been reported that EGCG inhibited ACC protein expression to decrease the DNL pathway and ATP production, thereby inducing lipogenesis depletion-associated apoptosis in colorectal cancer cells (211). Further, *in vivo* experiments found this inhibitory effect on the DNL pathway was closely related to the inhibition of Akt and ERK phosphorylation (211). A similar investigation also revealed EGCG suppressed ACC protein expression in hepatoma cells and proposed the potential mechanism of anti-DNL should be attributed to EGCG’s suppression on the receptor tyrosine kinases (RTKs), which mediate PI3K/Akt/mTOR complex 1 (mTORC1)/sterol regulatory element-binding protein 1 (SREBP1) axis (212). Additionally, it has been found that AMPK, a cellular energy level sensor that is activated when ATP decreases and ADP or AMP increases in cancer, mediated the dysregulation of ACC phosphorylation to subsequently inhibit ACC activity, thus attenuating the lipogenesis of hepatoma cells (213). Based on such a mechanism, an important study unveiled EGCG suppressed ACC activity through enhancing AMPK-mediated ACC phosphorylation, ultimately arresting the cell cycle or inducing apoptosis in human hepatoma cells (214).

FASN, another vital enzyme that regulates lipogenesis, is extremely low in expression and activity in almost all normal adult tissues but is dramatically overexpressed or activated in many cancer types, catalyzing the synthesis of acetyl-CoA and malonyl-CoA into long-chain fatty acids (205, 215). Over the past two decades, there has been considerable evidence supporting EGCG as an effective natural FASN inhibitor whose inhibitory effect is synchronized with the reduced endogenous lipid synthesis and the increased apoptosis (216, 217). According to previous studies, the primary cause for the inactivation of FASN may be determined by the irreversible reaction of EGCG with the β-ketoacyl reductase domain of FASN (217). Meanwhile, several researchers revealed EGCG attenuated EGF-induced FASN expression at protein and mRNA levels, suppressed Akt activation, and blocked the contact of transcription factor Sp-1 with its target gene in breast cancer cells, thus exerting its anti-proliferative pharmacological effects (218). Subsequently, these researchers extended their findings that EGCG attenuated EGFR 2 (HER2) or/and EGFR 3 (HER3)-induced FASN overexpression in breast cancer cells through suppressing PI3K/Akt signaling (219). HER2 overexpression and PI3K/Akt overactivation also determine increased sensitivity to FASN suppression-induced tumor cell death (215). Furthermore, the suppression of RTKs/PI3K/Akt/mTORC1/SREBP1 axis was also identified as a pathway by which EGCG downregulates FASN expression in hepatoma cells (212). Moreover, EGCG was found to inhibit mTOR signaling and downregulate FASN expression by stimulating AMPK expression in hepatoma cells (214). Taken together, it is not difficult to conclude that these findings support EGCG suppresses FASN expression through downregulating RTKs/PI3K/Akt/mTOR/FASN cascade signaling, thus disrupting DNL pathway in tumors (218, 220). Interestingly, a small sample-size randomized controlled study that examined the levels of FANS expression in prostate tissues of male patients undergoing prostate biopsy following oral EGCG capsule treatment showed that EGCG supplementation did not reduce FANS expression in the prostate (221). This may be due to the fact that short-term EGCG supplementation is insufficient to cause biologically significant changes in FASN levels in prostate tissues. Hence, extending follow-up time and increasing the homogenous patient population should be considered in future clinical trials.

Of note, EGCG’s inhibitory effects on fatty acid oxidation may not be as strong as it is on the DNL pathway. Numerous investigations have indicated EGCG has a slight to no inhibitory effect on the activity of carnitine palmitoyltransferase (CPT)-I, a vital rate-limiting enzyme in fatty acid catabolism, in comparison to the large inhibitory effect it had on FASN (222–224). Nevertheless, EGCG does cause the buildup of malonyl-CoA, which inhibits CPT action via acting as a regulator of CPT activity (212). Overall, the available evidence suggested that EGCG inhibited the DNL pathway without parallel stimulation of fatty acid oxidation.

Considering the pertinent studies from recent years, we have found targeting FASN with EGCG is advantageous to ameliorate the efficacy of anti-cancer therapy. Since EGCG can sensitize nasopharyngeal cancer to radiotherapy through inhibiting FANS, it may be developed as a radiosensitizer (225). Additionally, blocking FASN with EGCG has been found to significantly ameliorate the efficacy of cetuximab and pertuzumab in drug-resistant breast cancer, suggesting EGCG may synergize the antitumor effects of anti-EGFR and anti-HER2 therapies (226, 227). Even, inhibiting FANS expression using EGCG has been shown to significantly accelerate the differentiation of acute myeloid leukemia and re-sensitize all-trans retinoic acid-refractory tumor cells (228). Together, these findings have confirmed the feasibility of EGCG in combination treatment regimens.

### 4.5 Nucleotide metabolism

Nucleotides are critical substrates for a variety of anabolic pathways, particularly DNA and RNA biosynthesis processes (229). Due to the rapid growth and anabolism, tumor cells rely extensively on nucleotide synthesis pathways in addition to their large energy requirements (230). Recent research has demonstrated that enhanced nucleotide metabolism in tumors is strongly associated with many malignant behaviors, such as uncontrolled proliferation, chemotherapy resistance, immune evasion, and metastasis (231, 232). Intervention or modification of the dysregulation of nucleotide metabolism can promote tumor immunogenicity and increase antitumor immune response through disrupting purine and pyrimidine pools to increase mutagenicity and genomic instability (233).

With the development of metabolomics detection techniques, EGCG has been found to target tumor nucleotide anabolism. Metabolomic analysis of human colon cancer cells demonstrated EGCG affected nucleotide metabolic pathways and reduced the levels of uridine, uridine diphosphate (UDP), and ADP in tumor cells (234). Metabolomics results of lung cancer cells confirmed EGCG treatment significantly impaired the levels of adenine, cytosine, and 2-deoxycytidine in tumor cells (235). Similarly, our ongoing scientific investigation has also observed the regulatory effects of EGCG on purine and pyrimidine metabolic pathways in gastric cancer cells. Nevertheless, up to now, the mechanism by which EGCG interferes with *de novo* nucleotide biosynthesis in tumor cells has remained unclear. Based on the relevant findings, we are currently inclined to believe EGCG may block nucleotide biosynthesis through reducing the required feedstocks, including pentoses and one-carbon units. As previously mentioned, EGCG, as a PGAM1 inhibitor in glycolysis, was found to increase substrate 3-phosphoglycerate levels and decrease product 2-phosphoglycerate levels in tumor cells, thereby inhibiting 6-phosphogluconate dehydrogenase (G6PD) and leading to impaired pentose phosphate pathway (PPP) (190, 236). As a result, tumor cells may lack sufficient pentoses for nucleotide biosynthesis. In addition, glycine and serine are vital sources of one-carbon pool, where activated folate is the essential carrier. On the one hand, EGCG was found to increase the levels of glycine and serine in tumor cells, which might mean decreased consumption in their use for one-carbon unit production (235). On the other hand, EGCG was shown to effectively attenuate the activity of dihydrofolate reductase (DHFR) and disrupt the folate cycle, thereby diminishing the cellular production of nucleotides by damaging the one-carbon pool (237, 238). Collectively, the available evidence supports the inhibitory role of EGCG in nucleotide anabolism, which lays the foundation for the subsequent blockage of cancerous DNA and RNA synthesis.

The uninterrupted synthesis of DNA and RNA utilizing nucleotides is the most prominent proliferative property of tumor cells. In the past two decades, there has been ample evidence to confirm the direct intervention of EGCG and catechin derivatives on DNA and RNA synthases (239–241). One study revealed EGCG selectively suppressed mammalian DNA polymerase activity but it did not significantly suppress the comparable enzymes in plants and prokaryotes (242). This inhibition was observed to be competitive for the DNA template but non-competitive for the deoxyribonucleoside triphosphate (dNTP) substrate (243). However, a recent study has unveiled EGCG severely disequilibrates dNTPs in tumor cells, thus inhibiting DNA *de novo* synthesis and cell proliferation, which makes it difficult to resist the suggestion that EGCG may act as a ribonucleotide reductase inhibitor in tumor cells (244). Additionally, EGCG was reported to suppress RNA polymerase transcription from both exterior and internal promoters of genes and inhibit the protein expression and promoter activity of TFIIIB required for accurate RNA polymerase initiation in cervical cancer cells (240). Even more incredibly, it’s discovered that EGCG molecules bound to the double-stranded AG : CT oligomers of multiple nucleotide lengths, thus protecting the double-stranded DNA from being melted into single-stranded DNA, which subsequently leads to DNA replication disorders (245). These results fully confirmed EGCG can not only inhibit the anabolism of nucleotides but also prevent the synthetic transformation of nucleotides into nucleic acids.

Although we have concluded that EGCG regulates so many biological metabolic processes in tumors, whether there are other metabolic pathways controlled by EGCG remains unclear. Hence, abundant investigations are required to verify its regulatory effects and biological mechanisms on gluconeogenesis, mitochondria oxidative phosphorylation, glycogen metabolism, branched-chain amino acid metabolism, triglyceride metabolism, phospholipid metabolism, and cholesterol metabolism in tumors. In addition, its suppressive mechanism in PPP remains to be further revised and elaborated according to the results reported in the future literature. Corresponding *in vivo* animal experiments or organoid model experiments should also be implemented to systematically analyze EGCG’s effects on tumor metabolites and metabolic pathways.

## 5 Potential in anti-cancer immunotherapy

Immunotherapy, a biomedical milestone in cancer, has epoch-making significance. Several immunotherapies, such as immune checkpoint blockers, tumor vaccines, adoptive immunotherapies, and nanomedicine immunotherapies, have achieved durable antitumor responses, but their overall efficacy has been unsatisfactory (246). We summarized the application status and therapeutic effects of EGCG in the above immunotherapies, aiming to arouse its attention in immunotherapy, so as to achieve better clinical outcomes for cancer patients who undergo immunotherapy.

### 5.1 Anti-immune checkpoints

It’s well recognized that immune checkpoints including programmed death-1 (PD-1), PD-L1, and cytotoxic T lymphocyte antigen-4 (CTLA4) are mainly in charge of the immune escape of tumor cells under immune surveillance. The emergence of immune checkpoint inhibitors (ICIs) has revolutionized the treatment landscape for cancer, but only subset of patients can benefit from their immunotherapy (247). Therefore, innovative approaches are urgently needed to guide the development of novel immunotherapy combinations designed to maximize clinical benefits and survival outcomes.

Recent research has indicated that EGCG can play an antitumor immunological role by targeting PD-L1 molecule (248). Evidence suggested that unlike anti-immune checkpoint therapy, which simply obstructs PD-1/PD-L1 interactions, EGCG suppressed JAK/STAT signaling and downstream PD-L1 expression in melanoma cells, thereby reactivating CTLs to inhibit tumor cell growth (29). Further animal experiments revealed EGCG’s tumor inhibition was comparable to anti-PD-1 treatment. Another study indicated EGCG downregulated IFN-γ and EGF-induced PD-L1 expression in non-small cell lung cancer cells through suppressing JAK2/STAT1 and EGFR/Akt signaling pathways and boosted IL-2 expression in cancer-specific T lymphocytes (249). Additionally, due to EGCG’s “sealing effect” on multiple protein molecules, it may directly target the PD-L1 protein, thus disrupting the interaction between PD-1 and PD-L1. This possibility has been confirmed by molecular dynamics simulations in recent years. Relevant investigation revealed EGCG interacted directly and stably with the binding domain of PD-L1 dimer, in which non-polar interactions with pivotal residues played leading roles in their interactive binding (250). These findings suggest that EGCG may make ICIs more therapeutically effective and even may serve as an alternative approach to disrupt the PD-1/PD-L1 axis. Hence, EGCG in combination with ICIs seems viable as a novel therapeutic option. However, corresponding clinical studies are still needed to validate the abovementioned preclinical results. It is imperative to conduct large-sample and high-quality clinical trials to investigate the clinical benefits of EGCG in immunotherapy.

Of note, PD-1 and CTLA4 are mainly expressed on the T lymphocyte surface to put the brakes on the immune response. Based on the existing literature, it is unclear whether EGCG can inhibit the expressed levels of PD-1 and CTLA4 or interfere with their activities. However, other polyphenols may provide some reference for this role. For example, the natural polyphenol compound resveratrol was found to reduce the mRNA levels of PD-1 in pulmonary CD8^+^ T and CD4^+^ T cells, thus attenuating the lung metastasis of TNBC (251). Besides, the natural polyphenol curcumin has been revealed to reduce CTLA4 expression in Tregs, thereby inhibiting the immunosuppressive capacity of these Tregs (252). Given this, we highly expect EGCG to be endowed with similar anti-PD-1 and anti-CTLA-4 activities, and we believe that future studies will bear witness.

### 5.2 Nanomedicine-based immunotherapy

Currently, nanomedicine-based immunotherapy, a subdiscipline of immunology, is undergoing rapid development as the medium for researching innovative therapeutic strategies (253). With the great advancements of nanomedicine in immunotherapy, EGCG-containing nanoassemblies to ameliorate the efficacy of tumor immunotherapy have attracted wide attention. It was reported that a phenolic-based tumor-permeated nano-framework composed of EGCG and phenylboronic acid-modified platinum nanoparticles could not only downregulate PD-L1 expression but effectively promote the maturation of DCs and facilitate the infiltration of CTLs, thus amplifying immunotherapy outcomes and generating a powerful antitumor immune response in terms of the suppression of primary tumor and metastasis (254). An EGCG-delivery responsive penetrating nanogels based on a nano-sized controlled releasing system of the soluble cyclodextrin-drug inclusion complex was also found to decrease the PD-L1 expression in tumor cells, promote DC maturation, stimulate CTL infiltration and activation, and attenuate the suppression effects from Tregs, thereby switching “cold” tumor to “hot” tumor (255). Interestingly, a new delivery nanosystem of fluorinated-coordinative-EGCG was measured not only to substantially suppress PD-L1 expression but also to achieve perfect siPD-L1 delivery and improve siPD-L1 accumulation in tumor cells, thus providing a versatile vehicle for antitumor immunotherapy (256). Meanwhile, an MMP-2 activated nanoparticle carrying EGCG dimer and immune checkpoint B7-H3 (CD276) bispecific antibodies was reported to induce the elimination of glioblastoma through increasing the ferroptosis and strengthening the immune checkpoint blockade treatment (257). In addition, based on EGCG and ursolic acid, several scholars constructed a novel “core-shell” co-assembly carrier-free nanosystem that synergized immunotherapy of liver cancer through activating innate and acquired immunity (258). In conclusion, these EGCG-based nanoassemblies play pivotal roles in anti-immune checkpoint immunotherapy and enhanced antitumor immunological response. Nevertheless, the potential toxicity and internal metabolic pathways of these nanosystems remain unclear. Hence, some caution should be maintained regarding the safety of these nanomedicines, even if their immunotherapeutic effects are undeniable. Also, comparative studies on the immunological efficacy of different EGCG nanoassemblies remain not available, suggesting that nanosystem-based cancer immunotherapy is still a long way off.

### 5.3 Tumor vaccines

In fact, ICIs are often only effective in limited populations due to poor immunogenicity and inadequate infiltration or accumulation of CTLs in TME (259). How to break through the limitations of cancer immunotherapy is an urgent problem and challenge that modern medicine is facing. Clinical practice has demonstrated tumor vaccines can enhance the activation and proliferation of immune effector cells, so tumor vaccines are recognized as the next immunotherapy frontier (260, 261). There was increasing evidence that combined treatment with immunomodulatory doses of EGCG could enhance the immune response of specific CD8^+^ T lymphocytes and CD4^+^ Th1 lymphocytes induced by DNA vaccine, and provide long-term anti-cancer protection for treated mice, causing a more excellent cure rate compared to monotherapy (30, 262). Additionally, EGCG was confirmed to enhance antigen-specific immunotherapy efficacy of mesothelin-specific chimeric DNA vaccine by boosting the maturation of DCs, which is promising to offer an effective approach for immunotherapy in malignant mesothelioma, ovarian cancer, and pancreatic cancer (55). Recently, some researchers have prepared coordination microparticle vaccines utilizing EGCG and aluminum ions as ligands to wrap single tumor cells, which can be effectively internalized by DCs through endocytosis, thus improving the efficiency of immunological uptake (263). Further investigation found these microparticles coated with tumor cells effectively activated DCs and significantly increased Th1-related cytokine production, with immunotherapeutic effects comparable to those of polyinosinic-polycytidylic acid (PolyI:C) (264). This encapsulation strategy holds promise for personalized immunotherapy customized to individual patient’s cancer cells. Overall, EGCG, as an immunomodulator or an inhibitor of immune checkpoints, combined with tumor vaccines may be a valuable immunotherapy for tumor control.

### 5.4 Adoptive immunotherapy

Adoptive immunotherapy refers to the extraction, modification, and re-infusion of autologous or allogenic immune cells for therapeutic purposes (265). Antitumor immunotherapy with chimeric antigen receptor-modified T (CAR-T) cells has made tremendous progress and shown exhilarative clinical efficacy against hematological malignancies (266). Currently, the U.S. Food and Drug Administration has authorized five CAR-T immunotherapies for hematologic malignancies, however, the applicability of these immunotherapies in the field of solid tumors has lagged significantly (267, 268). Some of the major obstacles in solid tumors include inefficient CAR-T cell migration and infiltration, immunosuppressive TME, CAR-T cell manufacturing, lack of tumor-specific antigens, treatment-related toxicity, and antigen escape (267).

Since EGCG is able to improve the infiltration and CTL response and disrupt the inhibitory TME induced by immunosuppressive cells, it is ideally suited for adoptive immunotherapy in solid tumors. Several researchers prepared adoptive CD8^+^ T cells conjugated with immunotherapeutic liposomal drugs, containing EGCG, poly lysine, and the TLR9 agonist CpG (269). The results of the study found that this adoptive immunotherapy reduced the frequencies of MDSCs, Tregs, and M2 macrophages and elevated the proportion of CTLs in tumor-infiltrating lymphocytes, thus significantly improving the therapeutic efficacy in melanoma (269). Although the original intention of the investigators is that the inclusion of EGCG may facilitate the encapsulation efficiency and improve the stability of CpG, we consider its undeniable direct immunoregulatory roles in their CAR-T immunotherapy. Therefore, we recommend that immunotherapy studies combining CAR-T cells with EGCG be conducted as soon as possible to clarify its detailed effects in adoptive immunotherapy. In short, EGCG, as a potentially valuable companion to CAR-T immunotherapy, has a unique clinical application prospect that is expected to be translated into a treatment for solid tumors in the next few years.

## 6 Clinical application and limitation

Recently, ample preclinical observational studies have demonstrated green tea consumption is effective in reducing the risk of oral, prostate, breast, liver, gastric, and colorectal cancers to varying degrees (270–272). These results provide a theoretical basis for further clinical trials. A Phase II interventional clinical trial confirmed orally administered 800 mg or 1200 mg of EGCG once a day can dose-dependently downregulate the levels of oncology biomarkers, including proliferating cell nuclear antigen and clusterin, in patients’ bladder cancer tissues (273). Similarly, a daily intake of 400 mg of EGCG was also effective in diminishing serum prostate-specific antigen levels in men with precancerous prostate lesions (274). In addition, randomized controlled trials have unveiled EGCG can alleviate the formation of colorectal adenoma to some extent and remarkably attenuate NF-κB levels in colorectal tumor tissues (275, 276). Moreover, a clinical trial has revealed EGCG can reduce the percentage of mammographic density, a well-recognized predictor of breast cancer risk, in young women (277). Relevant evidence from a presurgical clinical study also supports a notable positive correlation between EGCG’s plasma levels and Ki-67 downregulation in breast tumor tissues (278). Overall, the above clinical studies have confirmed the biological potential of EGCG in antitumor therapy, which lays an important foundation for its further clinical application. Nevertheless, since these studies often lack further follow-up, it’s unclear whether EGCG treatment can ameliorate the therapeutic prognosis and clinical survival of tumor patients. More regrettably, there are still no clinical studies reported on EGCG combined with chemoradiotherapy and immunotherapy based on existing literature and clinical trial database.

Although abundant preclinical and clinical studies have confirmed the therapeutic potential of EGCG on tumors, it is undeniable that the oral bioavailability of EGCG is relatively low, which is an important stumbling block limiting its further clinical application (23). The possible reasons are that EGCG is mainly absorbed by passive diffusion, is prone to instability in alkaline intestinal fluid, and may be subject to catabolism by intestinal microorganisms, which may significantly affect its bioavailability (16, 17). Therefore, the development of liposomal encapsulation or nanomodification is conducive to ameliorating its stability and biological availability and achieving clinical efficacy beyond expectations. Additionally, other non-oral administration routes, such as intravenous and intramuscular injections, should also be refined in subsequent animal experiments and clinical studies for safety assessment and optimal dose selection. Notably, under the premise of low bioavailability, clinicians and researchers should be fully vigilant about its hepatotoxicity caused by the use of large doses while pursuing the improvement of therapeutic efficacy (279).

## 7 Conclusion

In conclusion, EGCG can boost the anti-cancer potential of cytotoxic effector cells and disrupt the functions of immunosuppressive cells and tumor-promoting cells, thus reactivating the antitumor immune response in TME. In addition, EGCG can target multiple upregulated metabolic reprogramming pathways, including glucose uptake, aerobic glycolysis, glutamine metabolism, fatty acid anabolism, and nucleotide synthesis. Moreover, EGCG, as an immunomodulator and immune checkpoint blockade, can enhance the efficacy of antitumor immunotherapy and may be a viable option for immunotherapy. Briefly, EGCG plays versatile regulatory roles in TME and metabolic reprogramming, which provides novel insights and combined therapeutic strategies for anti-cancer immunotherapy.

## Author contributions

DL: Conceptualization, Data curation, Visualization, Writing – original draft. DC: Conceptualization, Funding acquisition, Writing – review & editing. YS: Data curation, Visualization, Writing – review & editing. YC: Data curation, Visualization, Writing – review & editing. YZ: Writing – review & editing. JJ: Writing – review & editing. XC: Conceptualization, Funding acquisition, Writing – review & editing, Project administration.

## Appendix

**Table:**

67LR	67 kDa laminin receptor
α-KG	α-ketoglutarate
ACC	acetyl-CoA carboxylase
ACLY	ATP citrate lyase
ADCC	antibody-dependent cell-mediated cytotoxicity
ADP	adenosine diphosphate
Akt	protein kinase B
AMPK	adenosine monophosphate (AMP)-activated protein kinase
APCs	antigen-presenting cells
ARG1	arginase I
ASCT2	alanine/serine/cysteine transporter 2
ATP	adenosine triphosphate
bFGF	basic fibroblast growth factor
CAFs	cancer-associated fibroblasts
CAR-T	chimeric antigen receptor-modified T
CCL	C-C chemokine ligand
CTLs	cytotoxic T lymphocytes
COX-2	cyclooxygenase-2
CPT	carnitine palmitoyltransferase
CSF	colony stimulating factor
CTLA4	cytotoxic T lymphocyte antigen-4
CXCL	CXC chemokine ligand
CXCR	CXC chemokine receptor
D2HG	D-2-hydroxyglutarate
DCs	dendritic cells
DHFR	dihydrofolate reductase
DNL	*de novo* lipogenesis
dNTP	deoxyribonucleoside triphosphate
ECM	extracellular matrix
ECs	endothelial cells
EGCG	epigallocatechin gallate
EGC-M5	5-(3',5'-dihydroxyphenyl)-γ-valerolactone
EGFR	epidermal growth factor receptor
ERK	extracellular signal-regulated kinase
FAK	focal adhesion kinase
FASN	fatty acid synthase
FOXP3	forkhead box protein 3
G6PD	6-phosphogluconate dehydrogenase
G-CSF	granulocyte colony-stimulating factor
GDH	glutamate dehydrogenase
GLS	glutaminase
GLUT	glucose transporter
HCC	hepatocellular carcinoma
HER2	EGFR 2
HER3	EGFR 3
HGF	hepatocyte growth factor
HIF-1α	hypoxia-inducible factor-1alpha
HK	hexokinase
HSCs	hepatic stellate cells
ICAM-1	intercellular adhesion molecule 1
ICIs	immune checkpoint inhibitors
IDH	isocitrate dehydrogenase
IDO	indoleamine 2,3-dioxygenase
IFN-γ	interferon-gamma
IGF1R	insulin-like growth factor-1 receptor
IL	interleukin
iNOS	nitric oxide synthase
IR	insulin receptor
iTregs	induced Tregs
IRF-1	interferon regulatory factor 1
JAK	Janus kinase
LDH	lactate dehydrogenase
MAPK	mitogen-activated protein kinases
MDSCs	myeloid-derived suppressor cells
MHC	major histocompatibility complex
MHC-I	major histocompatibility complex class I
MMP	matrix metalloproteinase
MSCs	mesenchymal stem/stromal cells
mTOR	mammalian target of rapamycin
mTORC1	mTOR complex 1
MT1-MMP	membrane-type 1 MMP
NE	neutrophil elastase
NETs	neutrophil extracellular traps
NF-κB	nuclear factor-kappa B
NK cells	natural killer cells
nTregs	natural Tregs
NO	nitric oxide
NOX2	NADPH oxidase 2
PD-1	programmed death-1
PDGF	platelet-derived growth factor
PDGFR	PDGF receptor
PDH	pyruvate dehydrogenase
PDHA1	pyruvate dehydrogenase E1-α
PD-L1	programmed cell death ligand 1
PE	polyphenol E
PFK	phosphofructokinase
PGAM1	phosphoglycerate mutase-1
PGE2	prostaglandin E2
PI3K	phosphatidylinositol 3-kinase
PK	pyruvate kinase
PolyI:C	polyinosinic-polycytidylic acid
PPP	pentose phosphate pathway
RhoA	Ras homolog family member A
ROCK	Rho-associated kinase
ROS	reactive oxygen species
RTKs	receptor tyrosine kinases
Smad1	Smad family member 1
SNAT	sodium-coupled neutral amino acid transporter
SREBP1	sterol regulatory element-binding protein 1
STAT	signal transducer and activator of transcription
TAMs	tumor-associated macrophages
TANs	tumor-associated neutrophils
TCA	tricarboxylic acid cycle
TCR	T cell receptor
TECs	tumor-associated endothelial cells
TGF-β	transforming growth factor-beta
TIME	tumor immune microenvironment
TLR	toll-like receptor
TME	tumor microenvironment
TNBC	triple-negative breast cancer
Tregs	regulatory T cells
UDP	uridine diphosphate
UV	ultraviolet ray
VEGF	vascular endothelial growth factor
VEGFR	VEGF receptor
